# Participants’, caregivers’, and professionals’ experiences with a group-based rehabilitation program for Huntington’s disease: a qualitative study

**DOI:** 10.1186/1472-6963-14-395

**Published:** 2014-09-17

**Authors:** Jan C Frich, Merete Røthing, Alf Reiar Berge

**Affiliations:** Institute of Health and Society, University of Oslo, PO Box 1089, Blindern, N-0318 Oslo, Norway; Department of Neurology, Oslo University Hospital, N-0424 Oslo, Norway; Research Network on Integrated Health Care, Helse Fonna Local Health Authority, PO Box 2170, N-5504 Haugesund, Norway; Rehab-Nor, K. K. Lien Vei 50 I, N-4812 Kongshavn, Norway

**Keywords:** Rehabilitation, Huntington’s disease, Qualitative study, Patients, Caregivers, Health personnel

## Abstract

**Background:**

Research suggests that rehabilitation is beneficial for persons with Huntington’s disease (HD), but there is limited knowledge about participants’ experiences with residential rehabilitation programs. We therefore did a study to explore patients’, family caregivers’, and health professionals’ experiences with a group-based, residential rehabilitation program for individuals with early to mid-stage HD, focusing on three research questions: How did participants experience the structure and content of the program? What outcomes did patients experience? What challenges and success factors did health professionals report?

**Methods:**

Qualitative, explorative study, collecting data through in-depth interviews with nine family caregivers and 11 patients with early- and mid-stage HD, and focus group interviews with 15 health professionals. Data were analysed using systematic text condensation.

**Results:**

Some participants reported difficulties with defining individual rehabilitation goals, but written individualised plans and schedules were appreciated by all participants. Participants highlighted being member of an "HD-group" as a valuable experience, though tensions and conflicts could occur in groups. Participants typically reported improved gait and balance, increased self-confidence, and social benefits as outcomes. The intensive schedule was acceptable for most participants, but adjustments had been made to allow participants more time to eat, shower and dress between sessions. Success factors reported by health professionals were assigning every patient with a contact person, using clinical tests results to motivate patients, and supervising health professionals in patients’ local municipalities.

**Conclusions:**

Group-based residental rehabilitation was feasible for individuals with early- and mid-stage HD, and participants emphasised mental and social outcomes in addition to physical outcomes. The needs of persons with HD should be considerd when designing programs, to secure structure, continuity in personnel, and sufficient time between sessions.

## Background

Huntington’s disease (HD) is a hereditary autosomal neurodegenerative disorder caused by an expanded Cytosine-Adenine-Guanine (CAG) repeat in the HTT gene [1]. The disease is characterized by motor disturbances, psychiatric symptoms, and cognitive decline. Average age of diagnosis is 40–45 years, but symptoms have often been present for several years at the time of diagnosis [2]. Disease duration is commonly between 15–20 years, but symptom development and severity vary between individuals. Motor symptoms, such as gait and balance problems, are visible, but cognitive and behavioural changes are known to occur many years before clinical diagnosis [3]. Current treatment of individuals with HD consists mainly of symptom management and improving quality of life [2].

Research suggests that rehabilitation is beneficial for individuals with HD [4–8]. Observational studies indicate that multidisciplinary rehabilitation programs have positive effects on physical function, swallowing, balance, independence, mood, and social relationships [9–13]. A randomized study of a community-based exercise program found that the program was safe, feasible and acceptable, and suggests that structured exercise has benefits for persons with HD [14]. While adherence was a challenge in one inpatient rehabilitation program [9], patients’ adherence to a structured home based exercise program was high [10]. Zinzi et al. evaluated patients’ and caregivers’ experiences with an inpatient rehabilitation program [12] and found that caregivers reported gaining more knowledge of HD, a better sense of control and quality of life, empowerment in relations to doctors, and increased hope for their children at risk. Patients reported increased self-esteem and a sense of self-worth, and they emphasized being part of a community and establishing new significant relationships [12].

A multidisciplinary, intensive, group-based residential rehabilitation program for individuals with early to mid-stage Huntington’s disease (HD) was established in 2010 at two rehabilitation centres in Norway [13]. Patients were assessed and managed by a multidisciplinary team (Table 1), and a total of 31 out of 37 participants completed the program as planned. The one-year program consisted of three stays for three weeks in a rehabilitation centre, and patients were members of groups of four to six, and the program involved intensive physical training, information and support related to speech, nutrition, activities of daily living, and cognitive functions. Observational data suggest that the program is associated with improvement in balance, gait function, physical quality of life and reduced symptoms of depression and anxiety [13].

**Table 1:** **Professional groups and roles in the rehabilitation program**

Professional group	Role
Physician/neurologist	• Individual medical assessment at the beginning and the end of each stay
Nurses	• Coordination, observation and assistance to patients with impaired cognitive function or problems with ADL function
Physical therapist	• Focus on improvement of balance and gait function
	• Individually tailored training program based on each patient’s problems/strengths
	• Daily individual training for each patient, assisted by training assistants
	• Daily group activities such as training in groups in the gym and/or in a pool
Occupational therapist	• Focus on training of ADL, cognitive function, fine motor exercises and assessment of the need for assistive devices
	• Daily individual follow-up or group activities
Speech therapist	• Individual follow-up and group activities at least three times a week
Dietician	• Individual follow-up of patients’ with swallowing difficulties or low BMI
Social worker	• Individual counselling
	• Initiating processes of establishing an individual care plan
Psychologist	• Individual assessment and counselling

Process evaluation of complex interventions may add insights about users’ experiences, assess feasibility, and give input to future design, improvement of a program or interpretation of findings [15, 16]. Health professionals’ experiences may give valuable input to understanding barriers and facilitators for patients’ engagement in exercise programs [17]. As researchers with backgounds in neurology, nursing and health services evaluation and research, we had an interest in learning more about participants’ and professionals’ experiences with in-house rehabilitation. A number of issues and concerns that were raised by health professionals, patients and caregivers in the planning phase motivated the study: Did the clinical measures correspond with what patients and family caregivers experienced as important outcomes of the program? Would participants tolerate the intensive program and how would they accept the repeated clinical testing? With limited knowledge about these issues in the literature, we decided to do a study to explore participants’, caregivers’, and professionals’ experiences with the program, focusing on three research questions: How did participants experience the structure and content of the program? What outcomes did participants experience? What challenges and success factors did health professionals report?

## Methods

We found a qualitative and explorative approach most suitable, as such a design would allow program participants, caregivers and health professionals to articulate their own experiences and views without predefined categories. Interview guides (Table 2) for the individual interviews with participants, caregivers, and health professionals were developed by the members of the research team, based on existing literature and input from professionals and patients’ representatives involved in the programs.

**Table 2:** **Interview topics for participants/family caregivers and health professionals**

***Group***	***Topics***
Participants/family care givers	• Can you tell about your life at the moment?
	• How do you perceive your own health at the moment?
	• Do you have any problems related to movement, balance, nutrition or swallowing?
	• Do you experience any challenges with taking the initiative?
	• Do you experience any challenges with communication or behaviour?
	• How did you experience the stay at the rehabilitation centre?
	• What do you think about the information you got before the stay?
	• Do you feel that the professionals at the centre understood you?
	• Did you have a contact person?
	• Was the program tailored to your wishes and goals?
	• How did you experience the process of clarifying your goals?
	• How was goals evaluated during the course of the programme?
	• Were you involved in developing an individual plan?
	• What do you think about the content of the program?
	• What did you experience as most useful?
	• Has the program had any impact on your life after the stay?
	• What information were you given when you left the institution?
	• Was information sent to others?
	• What are your experiences with your municipality health care?
	• Can you please tell about any follow-up from the rehabilitation institution?
	• Do you experience any outcomes of the program? And if yes, what?
	• How can the centre improve the program?
Health professionals	• Can you explain your role in the program?
	• Can you describe the content of the program from your perspective?
	• Have you any previous experience with patients with Huntington’s disease?
	• How did you experience the planning phase?
	• What did the team do to map participants’ challenges with motor function, problems with swallowing, social function, and nutritional and behavioural issues?
	• Can you tell about processes to clarify participants’ goals for the stays?
	• Did you evaluate these goals?
	• Was an individual plan made?
	• What are your experiences with collaborating with participants’ municipalities?
	• How did you experience the program? What adjustments were made and what are the lessons learned?
	• How can the program be improved?

### Participants

Potential participants were approached through an invitation letter sent from the rehabilitation centres. Our sample was purposive and we aimed for variation regarding gender, age, functional decline, and disease stage. We recruited 11 individuals with early- and mid stage HD, six women and five men, who participated in the residential rehabilitation program. Two individuals with HD who were invited declined to participate in the study. Out of the 11 participants, six came from one institution and five from the other. Participants’ average age was 53 years (spread 34 to 63) and the sample consisted of persons with HD who had a variety of symptoms and functional challenges. We recruited nine caregivers, five men and four women. Half of the participants (six out of 11) and caregivers (four out of nine) were interviewed at two different stages during the program. Health professionals were invited to a focus group interview at the end of the program, and we conducted two focus groups with a total of 15 health professionals, 13 women and two men, from both centres. Various professionals’ backgrounds were represented in both focus groups, including physiotherapy, nurse, auxiliary nurse, speech therapist, behavioural therapist, psychologist, nutritional counsellor, occupational therapist, and social worker.

### Semi-structured interviews

Qualitative interviews [18] were conducted by JCF and ARB in study participants’ homes or at the rehabilitation centre. The interviews lasted between 30 and 70 minutes, were digitally recorded, and subsequently transcribed in verbatim. The interviews were loosely structured, aiming at an open dialogue, and the interview guide (Table 2) was used to check that topics were covered. Much effort was taken to allow participants and caregivers to respond and reflect freely and in their own words. Participants and caregivers were interviewed separately unless they wished to be interviewed together, and in some cases a patient with difficulties speaking was assisted by a caregiver. The interviews started with a general part, in which study participants were invited to tell about their background, before we explored their life situation at the moment, with a focus on challenges related specifically to living with HD. We thereafter explored their experiences with the program. The interviews were closed with an invitation to the interviewees to comment on potential improvements of the program.

### Focus group interviews

JCF moderated two focus group interviews [19] with health professionals who had been involved in the program. ARB was co-moderator of one focus group. The focus groups were loosely structured, using an interview guide (Table 2), exploring health professionals’ experiences with the program, their reflections and views about challenges and how such a program could be improved. The focus groups lasted between 60–70 minutes.

### Analysis

Data from the interviews were analysed by all authors using systematic text condensation, a procedure for thematic content analysis [20]. The analysis included four steps: (i) reading the material to obtain an overall impression and bracketing previous preconceptions; (ii) identifying units of meaning representing different aspects of experiences with the program, and coding for these; (iii) condensing and summarizing contents of each coded group; and (iv) making generalised descriptions regarding experiences with the program. All authors read through the transcripts, made lists of codes and met to negotiate and agree upon a coding frame. JCF coded all the transcripts manually and wrote a document with examples of quotes within each category. All authors reviewed and discussed the interpretation of the material until consensus was reached. Illustrating quotes were translated from Norwegian to English by the first author in the process of writing the article.

### Ethics

All participants were given verbal and written information about the study, and all participants signed an informed consent form. The project was approved by the Regional Ethics Committee, Health Region South-East (reference 2010/1026-1), Norway.

## Results

We have structured our findings in three main themes: Program structure and content, outcomes experienced by participants, and health professionals’ views on challenges and success factors. These themes are elaborated on in further detail below, with an indication of the source of illustrative quotes.

### Program structure and content

Participants were members of a "HD-group" who exercised together, met at meals and in the evening, and the comradery they developed with fellow group members was emphasised as important:

Meeting the group … we were six individuals in a team. It was so cosy. They had made a separate living room for us, and the rooms were next to each other in the corridor, so it was great (interview with participant).

While all participants underlined that being member of a group was a valuable experience, there were also reports about challenges and tensions in the groups, as illustrated in a comment by a participant:

She [another participant] was so … it was so noisy … we were tired, and none of us talked with her … we later got the opportunity to talk about it, and one of us asked a nurse: "Is it the disease that makes her talking all the time?" (interview with participant).

Most groups were mixed with regards to gender, and being the only woman or man in a group could be challenging, as stated by a women: "Men talk in a different way. They don’t talk like us women" (interview with participant).

Participants appreciated getting a written, individualised plan for each week, with a daily schedule for individual and group activities. While the schedule was feasible for most participants, there were participants who argued that the schedule had been too busy:

It was planned with a fixed schedule. That was okay, but when I had a swimming session, and it ended, there was not so much time … I did nearly not have the chance to dry my hair (interview with participant).

Individual rehabilitation goal setting was integral to the program, and one centre evaluated systematically the extent to which participants achieved their goals at the end of each stay. While some participants were easily articulated specific rehabilitation goals, others struggled to define goals, as illustrated in this quote:

Walk prettier. Walk faster … No I couldn’t come up with any other goals … Improve balance … it was stupid! How far are you from the target this time? Ten percent, twenty percent, thirty percent or forty percent … this time? (interview with participant).

One of the centres had implemented a system with a contact person assigned to each participant, which was appreacitated by participants and family caregivers as an arrangement that promoted continuity of care. Family caregivers suggested that the program could be improved if contact persons wrote a summary to participants at the end of each stay, so that family members could be updated on exercise programs and agreements that had been made.

### Outcomes experienced by participants

Participants reported experiencing physical, mental and social outcomes of the program, and the feeling that the situation was "stable" was viewed as an important outcome for some participants and families. A common experience among participants was improvement of gait and balance, which is illustrated in this statement: "My balance was much worse than it is today. It has improved after each stay, so that’s positive" (interview with participant). These experiences were echoed by family caregivers:

I think the balance has improved very much, and she has become more … not just sitting there in the sofa. It seems to me as if she has more energy (interview with family caregiver).

Participants reported improved swallowing, and better speech and memory. Participants and their close relatives did not always have similar views about outcomes, and participants usually reported greater effects than family caregivers. Participants emphasised improved self-confidence as a result of mastering specific tasks and being able to complete the program. One participant said:

I have become more open, in a way, and if I fall, I will get back on my feet again … this is not how it used to be. I used to be afraid of walking around, in case I would fall (interview with participant).

Participants and family caregivers emphasised that social outcomes were important, as underlined by one participant: "It’s important to meet people with the same illness. This is equally important as the exercise" (interview with participant). Patients underlined the significance of the social relationships they developed with fellow patients during the program:

I think it was a very social and good experience. I learned to know them all [members of the HD group]. We spent much time together and we talked about everything. I think this was the most important outcome for me (interview with participant).

Participants reported that the program also had a positive impact on social arenas outside the rehabilitation setting, as illustrated in this quote:

Clearly, this has been something she has experienced as very positive, and she has had something to talk about, which has had social spin-offs. The fact that the stays were long had an impact on her […] and something she could talk about to people. It has been positive from a social perspective, because her interests are restricted, and it’s limited what one can talk about, so in this respect it has had an effect, indirectly (interview with family caregiver).

Learning about other participants’ problems, and comparing oneself to others, could have a positive impact on individuals’ views about their own illness and living with HD, but some participants and caregivers reported worrying about the future after meeting individuals with a more severe illness. While some participants said that the program had not changed their views about HD, others experienced that meeting other people with HD had changed how they perceived themselves:

The disease is perhaps not as limiting as I first thought. I have been somewhat pessimistic about the impact of the disease … being together with ill patients helped me realize this (interview with participant).

### Health professionals’ views on challenges and success factors

Health professionals experienced that the program was feasible for most participants, but they had learned that some patients with HD needed more time compared with other groups. The time scheduled had been adjusted and individualised to allow for more time to eat, shower and dress between sessions. Some participants needed close follow-up due to a reduced ability to organise their daily activities, and sometimes health professionals had to go and look for participants who did not show up at a session. Health professionals also reported that goal setting could be challenging:

They [participants] did not always see a problem in what we thought ought to be a major issue for them […] but it’s their goal, and that’s how it should be, but if it’s not very concrete, I sometimes put words in their mouth, just to get something back (health professional in focus group interview).

Health professionals thought that their experience with goal setting for participants with HD was different compared with other groups of patients, and that participants’ cognitive impairment was a possible explanation. A physiotherapist shared the following reflection:

We usually base our approach on patients’ goals […] my experience with [the patients] I have met here is that it is difficult for them to specify what they need to work on. Goal setting for individuals with HD is not necessarily a straightforward process. Perhaps that’s exactly what they need to work on … they need to find out what they need to work on (health professional in focus group interview).

One success factor, according to health professionals, was assigning a contact person, usually a nurse or a physiotherapist, to each participant. The contact person’s role was to follow the participant from the beginning to the end of each stay, including meeting the participant when he or she arrived, communicating with family caregivers, coordinating multidisciplinary rehabilitation efforts, making weekly and daily schedules, and contacting health professionals in the participant’s local municipality. Even if the contact person was not always present, he or she represented an anchor for the patient and family caregivers. The contact person also had an important role in assessing the patient’s global needs:

When it comes to evaluating needs, it’s useful to see them over time, and then you learn more […] They may appear to be okay, they can do everything, they can participate in excursions, exercise, and then you realize that they aren’t able to cook ground beef (health professional in focus group interview).

The contact person at the rehabilitation centre had a role in supervising professionals in the patients’ local municipalities, documenting and articulating needs through phone calls and discharge reports. Family caregivers and patients reported that these initiatives had mobilised professionals and services locally.

Health professionals reported that clinical testing was well accepted by patients and that test results could be used to motivate patients. Some patients competed with their own scores and other patients’ scores, and that they had used test results to motive patients if the results showed signs of improvements:

They are interested in the physical tests, so I see this as positive element. You shouldn’t make too much out of it … and there’s an advantage with using many tests … You always find something positive to focus on … I think it’s important to keep this in mind with this diagnosis, that you think about how you present things, because I believe it may have a huge impact on motivation (health professional in focus group interview).

## Discussion

### Main findings

Our study suggests that some participants reported difficulties with defining individual rehabilitation goals, but written individualised plans and schedules were appreciated by all participants. Participants highlighted being member of an "HD-group" as a valuable experience, though tensions and conflicts could occur in groups. Participants typically reported improved gait and balance, increased self-confidence, and social benefits as outcomes. The intensive schedule was acceptable for most participants, but adjustments had been made to allow participants more time to eat, shower and dress between sessions. Success factors reported by health professionals were assigning every patient with a contact person, using clinical tests results to motivate patients, and supervising health professionals in patients’ local municipalities.

### Structure and content

We found that participants valued being member of a designated "HD-group", which is in accordance with findings in a previous study of residential rehabilitation in patients with HD [12]. Our study adds to previous knowledge about rehabilitation for persons with HD by documenting the potential for tensions and conflicts in groups. It is likely that some of these challenges could be determined by cognitive impairments and behavioural changes specific to individuals with HD. Health professionals should therefore be aware of such challenges in the group dynamics in a residential program for persons with HD.

We found that some participants reported difficulties with articulating specific rehabilitation goals, and that health professionals experienced that goal setting could be a challenging in HD. Goal setting is a core skill of rehabilitation professionals, and is considered as an essential component of any modern approach to rehabilitation [21, 22]. Studies suggest that patients’ experience of the rehabilitation process is significantly better, and that the nature of rehabilitation goals changes when patients are involved in goal setting [23]. Research demonstrate that goal setting processes may be complex and take time, and that patients’ ability to articulate goals need to be taken into account [24]. In a study of patients with diabetes mellitus, Mol [25] contrasts the "logic of choice" with the "logic of care", and argues that goals need to be developed as part of a process: "Within the logic of care, identifying a suitable target value is not a condition for, but a part of, treatment. Instead of establishing it before you engage in action, you keep on searching for it while you act." [25]. Our findings suggest a stepwise approach to goal setting in patients with HD. Health professionals may also consult caregivers and family members to establish meaningful rehabilitation goals for a participant. Our study indicates that individualised and written time schedules and plans help to establish clarity and structure, which is highly appreaciated by participants in the program. Persons with HD may have reduced ability to organise their own schedules [2, 3], and may therefore need clear information and reminders to be able to participate in a residential rehabilitation program.

### What outcomes do patients and caregivers experience?

The study was not designed to investigate objective clinical effects, but the findings indicate that there is concordance between particpants’ self-perceived outcomes, such as improved physical function, gait and balance, and objective clinical outcomes that have been reported elsewhere [9, 10, 13, 14]. Participants and caregivers in our study emphasised increased self-confidence and increased social participation, and these are outcomes that have been highlighted in previous research on rehabilitation programs for persons with HD [4, 12]. These social aspects may be central for HD patients’ motivation to participate in rehabilitation, but increased social participation in arenas outside the rehabilitation setting may also be an outcome to measure systematically in future research on rehabilitation in HD.

### Challenges and success factors

Our findings suggest that while an intensive residential rehabilitation program is feasible and acceptable for most participants, the institutions may have to make adjustments to accommodate specific needs of individuals with HD. A person with HD may need more time to eat, shower and dress between sessions than other groups of patients. A potential success factor reported in our study was assigning each patient with a designated contact person. Our results suggest that physical testing can be incorporated in the exercise and may be used to promote participants’ motivation.

In Norway, rehabilitation services can be provided at the local municipality level or in the specialised health service. While municipalities are responsible for primary health services such as nursing homes, home-based care, physiotherapy, and community-based rehabilitation, specialised rehabilitation services are organised by Regional Health Authorities. Increased coordination and collaboration between the two service levels is a political goal, and a coordination reform is being implemented to facilitate seamless transfers and integration between primary and specialised health care [26]. Our study suggests that specialised rehabilitation institutions, and the participants’ contact person, have the potential to supervise and guide local rehabilitation services and efforts. Specialised rehabilitation programs for patients with HD may thus secure long-term benefits if they plan for how needs assessments and rehabilitation plans can be fed back to the patient’s local health service.

### Methodological considerations

This explorative study was designed to investigate patients’, family caregivers’ and health professionals’ experiences with a group-based rehabilitation program. The number of participants in the study is limited, and it should be noted that patients who signed up for the rehabilitation program represent a self-selected group who are probably more motivated than the average person with HD. Our findings may thus not be valid for all patients with early- to mid-stage HD, but we think our sample is representative of the patients who choose to participate in an intensive residential rehabilitation program. The authors represent different professional backgrounds, two authors were involved in data collection, and all authors took part in the analysis of data. Also, using different sources of data, contrasting accounts of health professionals with those of patients and caregivers, we were able to reveal different stakeholders’ perspectives.

## Conclusion

Group-based residental rehabilitation is feasible for individuals with early- and mid-stage HD, and participants emphasise mental and social outcomes in addition to physical outcomes. The needs of persons with HD should be considerd when designing programs, to secure structure, continuity in personnel, and sufficient time between sessions.

## References

[1] Huntington’s disease Collaborative Research Group (1993). A novel gene containing a trinucleotide repeat that is expanded and unstable on Huntington’s disease chromosome. Cell.

[2] Novak MJ, Tabrizi SJ (2010). Huntington’s disease. BMJ.

[3] Paulsen JS (2011). Cognitive impairment in Huntington’s disease: diagnosis and treatment. Curr Neurol Neurosci Rep.

[4] Spring JA, Baker M, Dauya L, Ewemade I, Marsh N, Patel P, Scott A, Stoy N, Turners H, Viera M, Will D (2011). Gardening with Huntington’s disease clients – creating a programme of winter activities. Disabil Rehab.

[5] Bilney B, Morris ME, Perry A (2003). Effectiveness of physiotherapy, occupational therapy, and speech pathology for people with Huntington’s disease: a systematic review. Neurorehabil Neural Repair.

[6] Busse ME, Khalil H, Quinn L, Rosser AE (2008). Physical therapy intervention for people with Huntington disease. Phys Ther.

[7] Busse ME, Wiles CM, Rosser AE (2009). Mobility and falls in people with Huntington’s disease. J Neurol Neurosurg Psychiatry.

[8] Bohlen S, Ekwall C, Hellström K, Vesterlin M, Wiklund L, Reilmann R (2013). Physical therapy in Huntington’s disease – toward objective assessments?. Eur J Neurol.

[9] Zinzi P, Salmaso D, De Grandis R, Graziani G, Maceroni S, Bentivoglio A, Zappata P, Frontali M, Jacopini G (2007). Effects of an intensive rehabilitation programme on patients with Huntington’s disease: a pilot study. Clin Rehabil.

[10] Khalil H, Quinn L, van Deursen R, Dawes H, Playle R, Rosser A, Busse M (2013). What effect does a structured homebased exercise program have on people with Huntington’s disease? A randomized, controlled pilot study. Clin Rehabil.

[11] Thompson JA, Cruickshank TM, Penailillo LE, Lee JW, Newton RU, Barker RA, Ziman MR (2013). The effects of multidisciplinary rehabilitation in patients with early-to- middle-stage Huntington’s disease: a pilot study. Eur J Neurol.

[12] Zinzi P, Salmaso D, Frontali M, Jacopini G (2009). Patients’ and caregivers’ perspectives: assessing an intensive rehabilitation programme and outcomes in Huntington’s disease. J Public Health.

[13] Piira A, van Walsem MR, Mikalsen G, Nilsen KH, Knutsen S, Frich JC (2013). Effects of a one year intensive multidisciplinary rehabilitation program for patients with Huntington’s disease: a prospective intervention study. PLOS Curr.

[14] Busse M, Quinn L, Debon K, Jones K, Collett J, Playle R, Kelly M, Simpson S, Backz K, Wasley D, Dawes H, Rosser A, the Members of the COMMET-HD Management Group (2013). A ransomized feasibility study of a 12-week community-based exercise program for people with Huntington’s disease. J Neurol Phys Ter.

[15] Oakley A, Strange V, Bonell C, Allen E, Stephenson J, RIPPLE Study Team (2006). Process evaluation in randomised controlled trials of complex interventions. BMJ.

[16] Busse M, Khalil H, Brooks S, Quinn L, Rosser A (2012). Practice, progress and future directions for physical therapies in Huntington’s disease. J Huntingtons Dis.

[17] Quinn L, Busse M, Khalil H, Richardson S, Rosser A, Morris H (2010). Client and therapist views on exercise programmes for early-mid stage Parkinson’s disease and Huntington’s disease. Disabil Rehab.

[18] Bitten N (1995). Qualitative research: qualitative interviews in medical research. BMJ.

[19] Kitzinger J (1995). Qualitative research: Introducing focus groups. BMJ.

[20] Malterud K (2012). Systematic text condensation: a strategy for qualitative analysis. Scand J Public Health.

[21] Siegert RJ, Taylor WJ (2004). Theoretical aspects of goal-setting and motivation in rehabilitation. Disabil Rehabil.

[22] Van de Weyer RC, Ballinger C, Playford ED (2010). Goal setting in neurological rehabilitation: staff perspectives. Disabil Rehabil.

[23] Evans JJ (2012). Goal setting during rehabilitation early and late after acquired brain injury. Curr Opin Neurol.

[24] Holliday RC, Ballinger C, Playford ED (2007). Goal setting in neurological rehabilitation: patients’ perspectives. Disabil Rehabil.

[25] Mol A (2008). The Logic of Care: Health and the Problem of Patient Choice.

[26] Romøren TI, Torjesen DO, Landmark B (2011). Promoting coordination in Norwegian health care. Int J Integr Care.

[CR27] The pre-publication history for this paper can be accessed here:http://www.biomedcentral.com/1472-6963/14/395/prepub

